# Methylprednisolone treatment enhances early recovery following surgical decompression for degenerative cervical myelopathy without compromise to the systemic immune system

**DOI:** 10.1186/s12974-018-1257-7

**Published:** 2018-08-06

**Authors:** Pia M. Vidal, Antigona Ulndreaj, Anna Badner, James Hong, Michael G. Fehlings

**Affiliations:** 1 Division of Genetics & Development, Krembil Research Institute, University Health Network, Toronto, Ontario Canada; 2 Laboratory of Neuroimmunology, Fundación Ciencia & Vida, Santiago, Chile; 3 Institute of Medical Science, University of Toronto, Toronto, Ontario Canada; 4 Department of Surgery, Division of Neurosurgery and Spine Program, University of Toronto, Toronto, Ontario Canada; 5 Head, Spinal Program, Toronto Western Hospital, University Health Network, Toronto, Ontario Canada

**Keywords:** Degenerative cervical myelopathy, Methylprednisolone, Surgical decompression, Neuroinflammation, Immunosuppression

## Abstract

**Background:**

Degenerative cervical myelopathy (DCM) is caused by degenerative or congenital changes to the discs and soft tissues of the cervical spine, which leads to chronic compression of the spinal cord. The current treatment for moderate to severe DCM consists of surgical decompression, which, while effective in most cases, can result in neuroinflammation and spinal cord reperfusion injury, leading to perioperative neurological complications and suboptimal neurological recovery. The primary objective of this study was to assess, in a translationally relevant animal model of DCM, the efficacy of perioperative methylprednisolone (MP) in enhancing neurological recovery and to evaluate its effect on the inflammatory response following decompression.

**Methods:**

DCM was induced in C57BL/6 mice. Briefly, an aromatic polyether material was implanted underneath the C5-C6 laminae to cause progressive compression of the cervical spinal cord due to focal ossification. Decompressive surgery was undertaken at 12 weeks post initial biomaterial implantation. Animals received one dose of MP (30 mg/kg) or vehicle 30 min before decompression and at 2 weeks after decompression. Acute analysis of secreted cytokines and spinal cord microvasculature was complemented with immunohistochemistry for glial and neuronal cell markers. Locomotor outcomes were measured using the CatWalk system. The composition of circulating white blood cells was analyzed by flow cytometry.

**Results:**

A single dose of MP before decompression significantly sped locomotor recovery (*p < 0.05) and reduced the incidence of perioperative motor complications, without affecting the composition of circulating white blood cells. Histological assessment of the spinal cord showed significant neuronal preservation and a modest reduction in parenchymal inflammation.

**Conclusions:**

Our data suggest that MP reduces perioperative neurological complications following decompressive surgery for DCM by protecting neurons from inflammation, without compromising the composition of circulating immune cells. We propose that MP, which is commonly used for neurological disorders including spinal cord injury, be considered as a perioperative adjunct to decompressive surgery to attenuate neurological complications.

## Background

Degenerative cervical myelopathy (DCM) is an overarching term used to describe the most common forms of non-traumatic cervical spinal cord myelopathy (including cervical spondylotic myelopathy and ossification of the posterior longitudinal ligament). DCM, which increases in prevalence with aging, is caused by degenerative or congenital changes to the discs and soft tissues of the cervical spine, which leads to chronic compression of the spinal cord [1]. Importantly, increased recruitment and excessive activation of different immune cells (activated microglia/macrophages, T cells and neutrophils), accompanied by the production of inflammatory cytokines in the spinal cord, have been shown to contribute to the progression of DCM [2, 3].

DCM is associated with significant neurological dysfunction, including gait impairment, loss of manual dexterity, and pain [1]. The current treatment, particularly with moderate to severe impairment [4], consists of surgical decompression [5]. However, approximately 4% of patients who undergo decompression develop perioperative neurological complications, including worsening of myelopathy and delayed C5 palsy [5]. Moreover, while most patients show neurological recovery with decompressive surgery, approximately 20% of patients fail to show neurological improvement, with a minority exhibiting continued neurological decline. Additionally, our DCM mouse model has demonstrated that post-decompression neurological decline is associated with the presence of an ischemia-reperfusion injury (IRI) and increased activation of the immune system [6, 7]. Thus, neuroprotective or neuroregenerative strategies, which complement surgical decompression and rehabilitation approaches, would enhance the management of patients with DCM.

In the present study, we hypothesized that reducing neuroinflammation following decompression for DCM would attenuate perioperative neurological decline and promote enhanced neural recovery. To examine this hypothesis, we used a mouse model of DCM that is associated with severe neuroinflammation [7]. This model is intended to mimic patients who present with chronically progressive DCM. The anti-inflammatory treatment selected for this study was methylprednisolone (MP), which has long-standing use in clinical practice as an anti-inflammatory and neuroprotective treatment for traumatic spinal cord injury (SCI) [8]. Patients with cervical SCI and low baseline severity of injury have been shown to benefit the most from MP treatment [9]; however, the use of MP for SCI has been questioned by some clinicians due to heterogeneous findings reported in the literature [10]. In animal models of SCI, MP has also been shown to preserve neurons and limit axonal dieback, as well as reduce microglia/macrophages and cytokine levels immediately following injury [11]. In addition to its use in SCI, the use of perioperative corticosteroids as a complementary approach to decompressive surgery has been shown to reduce pain as well as the duration of postoperative hospitalization in patients with lumbar and cervical radiculopathy due to degenerative conditions [12, 13]. Given this background, we sought to examine the repurposing of MP as a potential neuroprotective treatment for DCM as a complement to surgical decompression.

In the present study, we examined the effectiveness of MP treatment to reduce inflammation following decompression in a mouse model of DCM at the C5-C6 level. We observed perioperative MP treatment accelerated locomotor recovery by preserving the number of neurons, while modest effects on inflammation were observed. There was also a reduction in the incidence of perioperative motor complications (defined as reduced ankle movement and plantar stepping, forepaw palsy, and upper/lower limb stiffness and/or weakness) following MP treatment. Importantly, no harmful side effects (including increased incidence of wound infection and death) or changes in the peripheral white blood cell composition were observed after MP treatment.

## Methods

### Animals

The Animal Use Committee at the University Health Network (UHN; Toronto, Canada) approved the study protocol, and experiments were carried out in accordance with the committee recommendations. Adult 8-week-old female C57BL/6 mice were purchased from the Ontario Council Institute (Canada) for use in this study. Investigators remained blinded to the treatment groups for the duration of the study.

### Spinal cord compression and decompression

DCM was induced in mice as previously described [7]. Briefly, an aromatic polyether material was implanted underneath the C5-C6 laminae to cause chronic and progressive compression of the cervical spinal cord due to focal ossification. This compression model mimics ossification of the ligamentum flavum, one of the known causes of DCM [1]. At 12 weeks post-compression (i.e. post-material implantation), mice underwent decompression using a microdrill to remove the osteoid formation between the aromatic polyether and laminae. All surgical procedures were performed under anesthesia using 2% isoflurane. Following deep anesthesia with isoflurane, animals were sacrificed at 24 h, 2 weeks, or 5 weeks after surgical decompression.

### Experimental groups

**Fig. 1:**
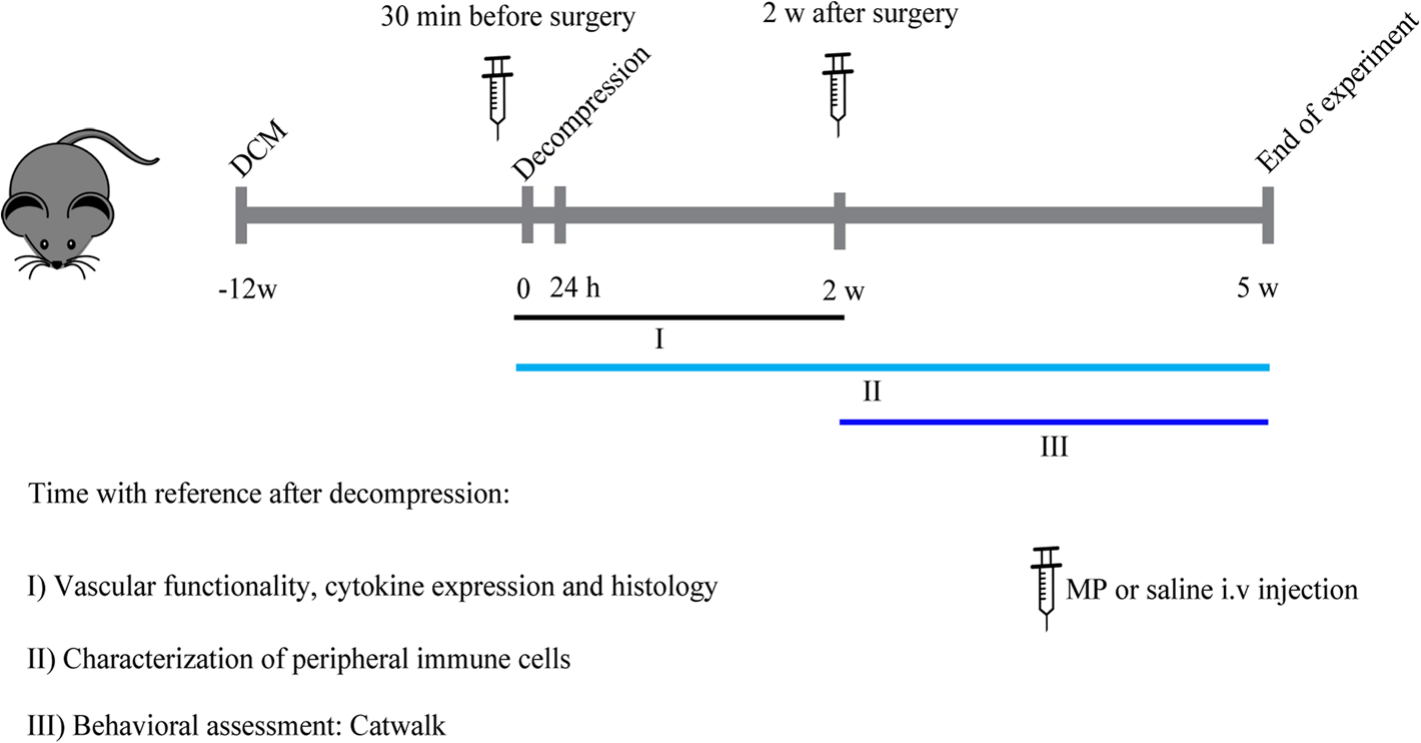
Scheme of the experimental design. The two time points selected for MP or saline (i.v) injection are depicted along with the readouts used. A color code was used to indicate the readouts used at selected time points. Animals were followed for 5 weeks after decompression

Animals were decompressed at 12 weeks following compression and randomly assigned to one of two experimental groups: (1) decompression with saline treatment (herein referred to as saline) and (2) decompression with MP treatment (30 mg/kg). MP treatment was given intravenously (i.v.) 30 min before surgical decompression and 2 weeks following decompression (Fig. 1). A second cohort of animals that did not undergo decompression (naive group) was used to assess the effect of decompression on the peripheral immune cell composition at 2 and 5 weeks following decompression.

### Automated gait analysis

The CatWalk XT 10.6 system (Noldus, The Netherlands) was used to assess locomotion of DCM and decompressed animals, as described previously [7]. Stance phase, stride length, and swing speed were analyzed in both forepaws and hindlimbs at 2 and 5 weeks following decompression. Runs (2–3 per animal) were averaged and included in the analysis if they had a duration between 0.50 and 5 s, without significant differences in speed between runs [7, 14]. No food restriction or reward was used to motivate mice to perform the task.

### Blood collection and flow cytometric analysis

Repeated blood sampling of mice was performed via saphenous vein puncture without anesthesia. Blood samples were collected using a 23G needle with ethylenediaminetetraacetic acid (EDTA)-coated tubes to avoid blood coagulation [15] and were analyzed by flow cytometry [16]. Samples were collected at 12 weeks of DCM, and at 24 h, 2 weeks, and 5 weeks after decompression. Red blood cells where lysed in red blood cell lysis buffer [7] and washed twice with phosphate-buffered saline (PBS). Cells were first stained with viability dye (Fixable viability dye eFluor 780, Thermo Fisher Scientific) for 20 min, followed by extracellular staining with fluorescent antibodies to distinguish granulocytes, monocytes, and T cells in the blood. The antibodies used were as follows: Ly6C-Pacific blue (clone HK1.4; BioLegend), Ly6G-PerCP/Cy5.5 (clone 1A8; BioLegend), CD11b- FITC (clone M1/70; BioLegend), CD3-PE (clone 17A2; BioLegend), and CCR2-PE (clone 475301; R&D Systems). Matching isotype controls were used to set the gates during data acquisition and analysis. Data were acquired using a BD LSR II flow cytometer (BD Biosciences) and analyzed using FlowJo X 10 (Trestar).

### Luminex assay

Cervical spinal cord homogenates from a 0.3 cm long section of tissue centered at the compression area were prepared following transcardial perfusion with PBS, as previously described [7]. Levels of selected cytokines (IL-1α and β, TNF-α, IL-4, IL-10 and IL-6) were measured using a mouse cytokine array at 24 h after decompression (Eve Technologies, Calgary, AB, Canada).

### Immunohistological analysis of the spinal cord

Animals were transcardially perfused with PBS, followed by 4% paraformaldehyde (PFA) in PBS. The spinal cords were dissected out (0.6 cm long section centered at the compression epicenter), post-fixed, and cryoprotected in 30% sucrose/PBS for 48 h. Coronal sections (30 μm thick) were prepared and blocked (10% non-fat milk, 1% BSA, 0.3% triton X-100 in PBS) for 1 h at room temperature (RT). Incubation with primary antibody was performed overnight (at 4 °C), followed by 1 h incubation with 4′,6-diamidino-2-phenylindole (DAPI; 1:200) and the corresponding secondary antibody at RT. The following primary antibodies were used: NeuN conjugated to Alexa-Fluor 555 (1:250; clone A60, Millipore), Iba1 (1:300, Wako, 019-19741), GFAP-conjugated to Cy3 (1:300, Sigma-Aldrich, MAB3402C3), and Olig-2 (1:500; AB9610, Millipore Sigma). NeuN^+^ cells were automatically quantified over a 3240 μm area centered at the lesion epicenter in gray matter using the cell counter plug-in from ImageJ, whereas Iba1^+^ cells were manually counted in the dorsal and ventral horns. The area of the dorsal and ventral horns evaluated for GFAP immunoreactivity was traced using ImageJ software in a constant square region of 89 × 89 μm, as described before [7]. Olig-2 area in the gray matter was automatically quantified using a customized script and normalized to the area of DAPI staining, as a reproducible measure of Olig-2 cells accounting for background signal and overlapping cells. All images were acquired using either a × 10, × 20, or × 40 objective lens with a Nikon eclipse Ti C2+ inverted confocal microscope with the NIS element imaging software version 4.20.

### In vivo power Doppler imaging

The spinal cord microvasculature was assessed with power Doppler imaging at 24 h and 2 weeks after surgical decompression, as previously described [17]. Static field of views (20 sagittal slices) that encompassed the entire lesion (130 × 90 px) were cropped from each sagittal stack and batch cropped in Photoshop CS6™. The resulting cropped images were then thresholded for the Doppler signal and batch-measured in ImageJ software using a customized script. The Doppler area of each sagittal slice was computed by taking the product of the total area of each image and the percent area of Doppler signal. The sum, termed the total Doppler area (TDA), was used to reflect the total functional vascularity of each sagittal stack.

### Statistical analysis

The results were analyzed using Prism 5.0 (GraphPad, La Jolla, CA, USA) and SPSS version 22 (IBM, Armonck, NY, USA) software. Histology, Doppler, and Luminex results comparing two groups were analyzed using a *t* test. Flow cytometry and CatWalk results were analyzed using either a one or two-way analysis of variance (ANOVA) with Tukey’s post-hoc test. All data are presented as mean ± standard error of the mean (SEM). Results were considered significant at a *p* value ≤ 0.05.

## Results

### Methylprednisolone mitigates perilesional spinal cord inflammation

**Fig. 2:**
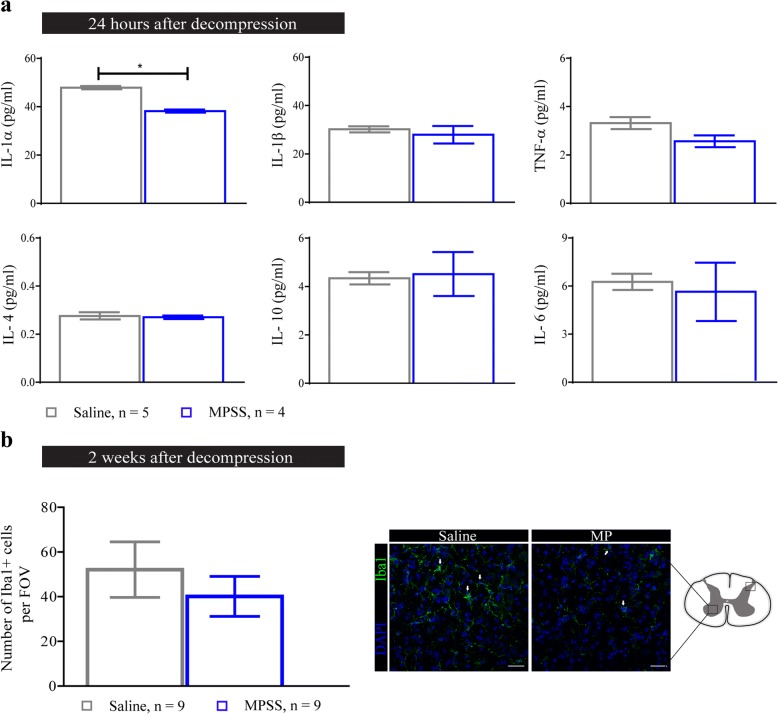
Methylprednisolone treatment diminishes acute cytokine production. **a** Expression of selected inflammatory and anti-inflammatory cytokines was measured in the spinal cord at 24 h after decompression using a Luminex assay. Overall, MP treatment significantly decreased the levels of IL-1α (*p < 0.05). **b** Immunohistological analysis of the number of Iba1^+^ cells around the C5-C7 region at 2 weeks after decompression. Their number in the dorsal and ventral horns showed a non-significant reduction after MP treatment. Representative images for each treatment group are shown as well as a schematic of a spinal cord depicting the area analyzed. The white arrows indicate Iba1+ cells in the spinal cord. Data were analyzed using an unpaired Student’s *t* test and are presented as mean ± SEM

We designed a randomized, blinded experiment where animals were divided into two groups that received two i.v injections of either MP or saline at two different time points, as shown in Fig. 1. We focused our assessments during the first 24 h and 2 weeks following decompression due to the development of IRI and high activation of the immune system observed in our animal model around these two time points [6, 7]. Although MP treatment has been shown to reduce inflammation after traumatic SCI [11], at 24 h after decompression in DCM the production of inflammatory cytokines (IL-1α, IL-1β, and TNF-α) was only modestly reduced in the MP group (*n* = 4), and only reached significance for IL-1α (Fig. 2a) compared to the saline treated group (*n* = 5). The expression of cytokines with anti-inflammatory (IL-4 and IL-10) and pleiotropic functions (IL-6) was not affected by MP treatment (Fig. 2a). Previous studies have demonstrated MP’s immunosuppressive effects by showing reduced macrophage/microglia proliferation following traumatic SCI [18]. For this reason, we quantified the number of Iba1^+^ cells in the spinal cord at 2 weeks after decompression. MP treatment slightly reduced the number of Iba1^+^ cells in the gray matter compared to the saline-treated group (*n* = 9 for both groups; paired *t* test, *p* = 0.14); however, this was not significant (Fig. 2b).

### Glial cell recruitment is not altered by methylprednisolone treatment after decompression

**Fig. 3:**
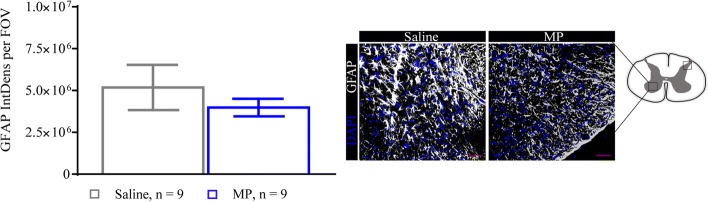
Astrogliosis is not significantly affected by MP treatment. GFAP immunoreactivity in the dorsal and ventral horns was analyzed around the C5-C7 region at 2 weeks after decompression. A non-significant reduction in GFAP immunoreactivity was observed after MP treatment. Representative images for each treatment group and a spine schematic depicting the area analyzed are shown. Data were analyzed using an unpaired Student’s *t* test and are presented as mean ± SEM. Scale bar = 25 μm. FOV, field of view

The immunosuppressive effects of MP have been shown to alter GFAP expression following traumatic SCI [19]. However, at 2 weeks after decompression, MP treatment (*n* = 9) only slightly reduced astrogliosis in the gray matter compared to the saline-treated group (*n* = 9), but these differences were not significant (Fig. 3).

### Early functional vascularity is not altered by methylprednisolone

**Fig. 4:**
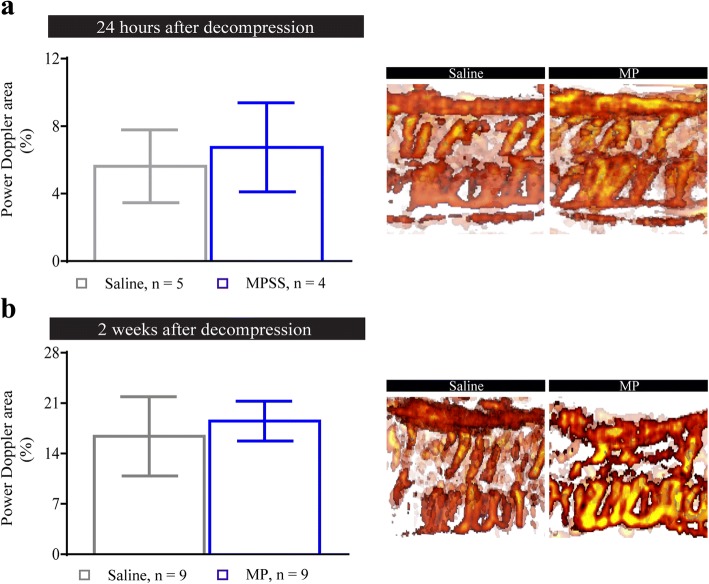
Methylprednisolone treatment does not affect functional vascularity. **a**, **b** Functional vascularity was measured using power Doppler at 24 h (**a**) and 2 weeks (**b**) after decompression. No significant changes were observed at any analyzed time points between the treatment groups. Data were analyzed using an unpaired Student’s *t* test and are presented as mean ± SEM

Vascular compromise and neuroinflammation have been implicated in the progression of DCM [7, 20, 21]. To examine this, we measured functional vascularity using Power Doppler imaging at 24 h (saline *n* = 5; MP *n* = 4) and 2 weeks (*n* = 9 for both groups) after decompression. At both time points, no significant differences between MP and saline treated groups were observed (Fig. 4a, b). Interestingly, vascular function (at 2 weeks) showed increased recovery compared with the 24 h assessment for both MP and saline groups (Fig. 4a, b).

### Reduced number of circulating white blood cells after decompression

**Fig. 5:**
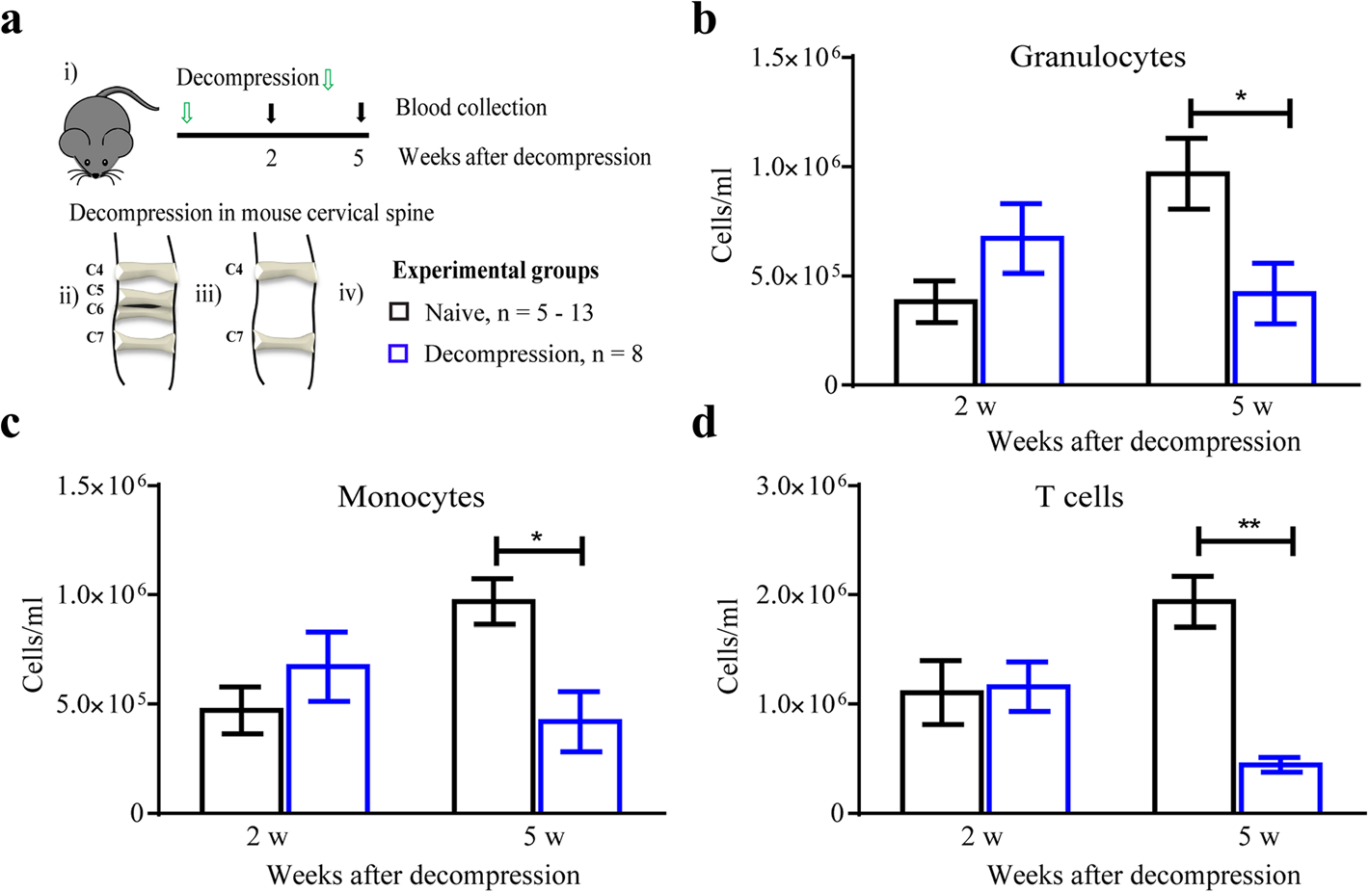
Peripheral white blood cell numbers decrease after decompression. **a** Scheme of the time points assessed for blood collection based on weeks after decompression (i), the bony formation between the material and laminae was removed at 12 weeks after implantation to achieve decompression (ii–iii), and summary of the experimental groups (iv). At 5 weeks after decompression, the number of all cell types (**b**–**d**) was significantly reduced (*p < 0.05, **p < 0.01), without significant changes observed at 2 weeks. Data were analyzed by a two-way ANOVA and are presented as mean ± SEM of 3–4 independent experiments

Trauma or surgery can induce a stress response, which encompasses changes in both the endocrine and immune system [22, 23]. In order to characterize the changes in the circulating immune cells following surgery, we compared the composition of white blood cells in decompressed and age-matched naive animals at 2 and 5 weeks after decompression (Fig. 5a). At 2 weeks after decompression, there were no significant differences in monocytes, granulocytes, or T cell numbers (Fig. 5b–d). However, at 5 weeks after decompression, the number of all cell types was significantly reduced in decompressed animals (*n* = 8) compared to age-matched naive animals (*n* = 3–8) (Fig. 5b–d).

### Methylprednisolone does not compromise white blood cell composition

**Fig. 6:**
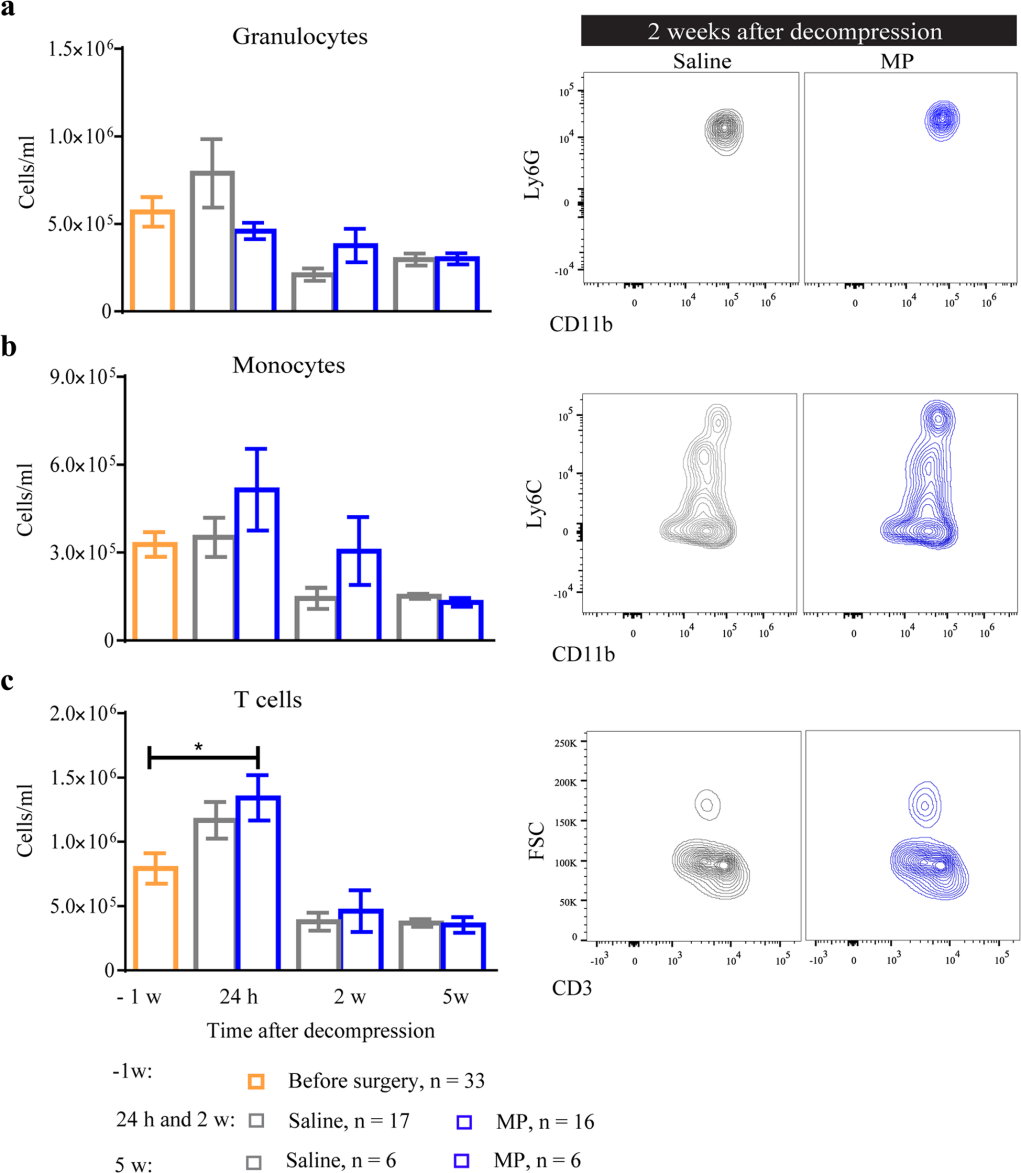
Methylprednisolone treatment does not affect the number of peripheral white blood cells. Blood samples were longitudinally collected at − 1 week, 24 h, 2 and 5 weeks after decompression and assessed by flow cytometry. The number of granulocytes (**a**), monocytes (**b**), and T cells (**c**) was not affected by MP treatment compared with the saline group. Representative flow charts for each cell type at 2 weeks after decompression are shown with quantification. Data were analyzed using two-way ANOVA and are presented as mean ± SEM

The immunosuppressive action of steroids, especially MP, has generated concern regarding the increased risk of infections, particularly when administered in conditions where the peripheral immune system is already suppressed, such as traumatic SCI [24, 25]. Since we did not find any signs of peripheral immune suppression following decompression, we hypothesized that systemic administration of MP early after decompression would not compromise the peripheral immune response. To examine this, we quantified the number of granulocytes, monocytes, and T cells before (*n* = 33) and after MP treatment with flow cytometry. At 24 h after decompression, the number of granulocytes and monocytes was not significantly altered by either decompression alone or between the MP and saline treatments (Fig. 6a, b). Further, the number of T cells was not altered by MP treatment compared with the saline-treated group. However, MP increased the number of T cells compared with levels before decompression (Fig. 6c, *p < 0.05). At 2 weeks after decompression, the number of granulocytes, monocytes and T cells was reduced compared with the 24-h assessment. Granulocytes and monocytes were slightly elevated in the MP-treated group compared with the saline group, without reaching significance (Fig. 6a, b). No significant changes were reported in any of the above white blood cell populations at 5 weeks after decompression (Fig. 6).

### Methylprednisolone treatment leads to improvements in early locomotor outcomes after surgical decompression

**Fig. 7:**
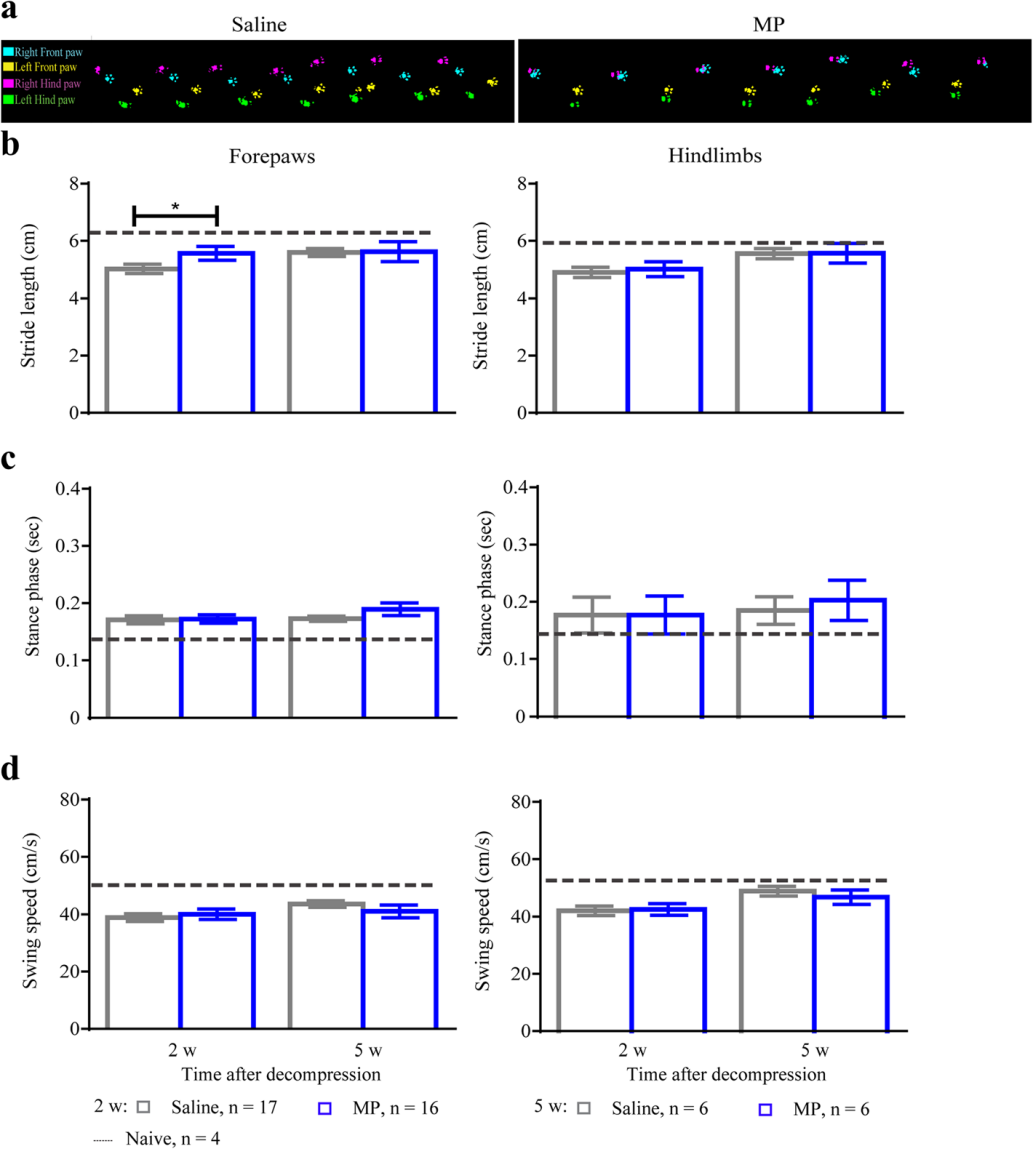
Methylprednisolone treatment speeds recovery of gait. Locomotor recovery was quantified using the CatWalk system at 2 and 5 weeks after decompression. **a** Representative footprints of saline- and MP-treated animals at 2 weeks after decompression are shown. **b** Stride length in the forepaws was only significantly improved at 2 weeks after decompression, without significant changes at 5 weeks. No significant changes were observed in the hindlimbs (**c**–**d**). Stance phase and swing speed were not significantly affected in the forepaws and hindlimbs of the two treatment groups at any analyzed time point. Dotted line represents naive non-injured animals. Data were analyzed using a two-way ANOVA and are presented as mean ± SEM

Gait impairment is one of the first symptoms to present in patients with DCM [26]. We assessed changes in select forepaw and hindlimb gait parameters using the CatWalk system in DCM animals (*n* = 33) at 2 and 5 weeks after surgical decompression. One week before surgical decompression (pre-decompression baseline), no significant differences were observed in stance phase, swing speed, and stride length between DCM animals (data not shown). At 2 weeks after surgical decompression, we observed that animals treated with MP had a walking pattern similar to non-injured animals in contrast with the saline group that showed persistent deficits (Fig. 7a). Interestingly, MP treatment sped the temporal recovery of stride length as compared with the saline-treated group (Fig. 7b, *p < 0.05), but it did not affect stance phase (Fig. 7c) and swing speed (Fig. 7d). Nevertheless, the improvement in stride length did not persist at 5 weeks after decompression, where gait parameters reached similar values to the naive non-injured animals (*n* = 4) (Fig. 7b–d). These results suggest that the first dose of MP might have an early neuroprotective effect that enhances recovery of some locomotor parameters after decompression, without long-term effects. Of note, there was a reduction in the incidence of postoperative locomotor complications following decompression after MP treatment (Table 1). Specifically, at 24 h after surgery, 16.7% (3 out of 18) of the animals receiving the control treatment presented with motor complications (reduced ankle movement and plantar stepping and upper or lower extremity stiffness), whereas only 5.5% (1 out of 18) of animals treated with MP showed similar complications (*p* = 0.3).

**Table 1:** Motor complications after decompression

Groups	Day 1	No complications
Decompression + saline	3 animals (16.7%)	15 animals (83.3%)
Decompression + MP	1 animal (5.5%)	17 animals (94.5%)

### Methylprednisolone treatment induces neuronal cell preservation

**Fig. 8:**
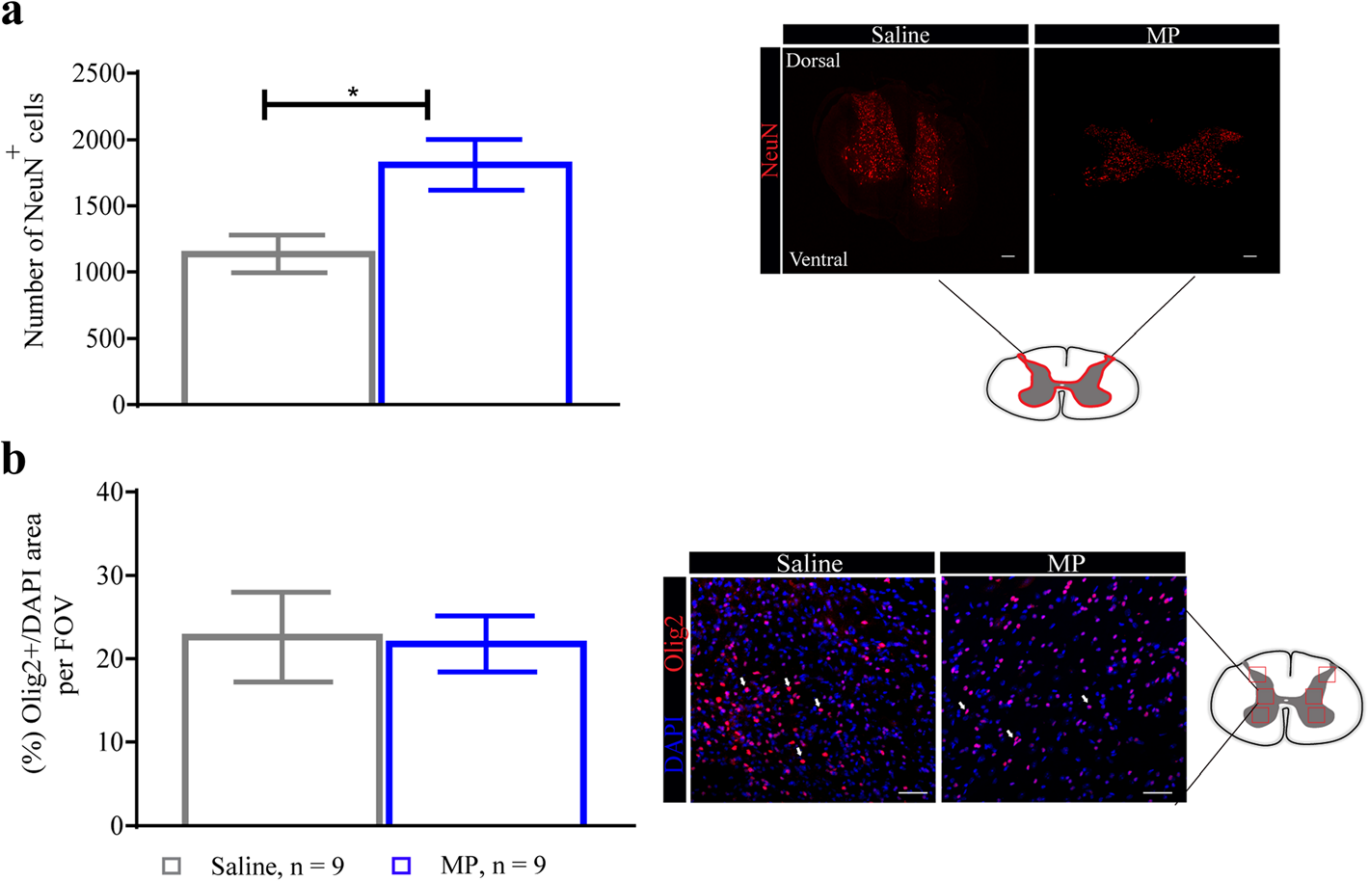
Methylprednisolone treatment promotes neuronal preservation in DCM. Immunohistochemical analysis of the decompressed spinal cord around the C5-C7 region. **a** Number of NeuN^+^ cells and representative images of the two treatment groups. NeuN^+^ cells were significantly increased compared with the saline-treated group at 2 weeks after decompression. **b** The percentage of Olig2^+^/DAPI^+^ area was not significantly different between the two treatment groups. Data were analyzed using an unpaired Student’s *t* test and are presented as mean ± SEM. Scale bars = 25 μm (**a**) and 500 μm (**b**). The arrows in **a** and **b** indicate a positive cell for each staining. FOV, field of view

Chronic compression of the cervical spinal cord leads to a reduction in blood flow to the cord and thereby results in the loss of spinal cord motor neurons [6, 7, 27]. In order to assess neuronal preservation, we used the neuronal markers NeuN and Olig-2 to quantify the number of positive cells around the C5-C7 region using immunohistochemistry. Our results show MP significantly preserved the number of NeuN^+^ cells in the spinal cord compared with the saline-treated group at 2 weeks following decompression (*n* = 9 for both groups) (Fig. 8a). Furthermore, oligodendrocytes are known to undergo apoptosis during the progression of DCM [3], but are protected after MP treatment [28]. However, peri-operative treatment with MP did not alter the expression of oligodendrocytes compared with the saline-treated group at 2 weeks after decompression (Fig. 8b).

## Discussion

In this study, we showed that perioperative MP treatment following decompressive surgery for DCM accelerates locomotor recovery through enhanced neuronal preservation and reductions in inflammation, without compromising the composition of peripheral immune cells populations. Traumatic SCI has been shown to elicit systemic and parenchymal activation of the immune response that can contribute to organ dysfunction and post-injury complications [29] [25]. However, it is not known whether DCM, the most common form of non-traumatic SCI, has similar immune system activation. Therefore, we addressed the effectiveness of a combinatorial treatment paradigm to target delayed DCM decompression surgery. Previously, our lab has shown successful repurposing of the sodium-glutamate blocker Riluzole, an FDA-approved drug for the treatment of amyotrophic lateral sclerosis, in mitigating ischemia-reperfusion injury in an experimental animal model of DCM [6]. This work was the foundation for the CSM-PROTECT clinical trial aimed at evaluating the efficacy of Riluzole in combination with decompression [30]. However, considering significant inflammation associated with delayed decompression, this study (for the first time) assessed the potential combinatorial use of the anti-inflammatory drug MP. Overall, MP treatment sped locomotor recovery and enhanced neuronal preservation, without compromising peripheral white blood cell composition.

Major surgical procedures can lead to alterations to the hemodynamic, endocrine, and immune functions of the body. Specifically, this leads to an initial activation of the peripheral immune system along with enhanced blood flow to muscle, liver, and ischemic organs [22]. These changes are followed by a phase of depressed immune function and recovery [31]. It is important to understand the duration of these phases following surgical decompression in order to determine the optimal time window and route (e.g., systemic or local) of administration for potential peri- and/or postoperative treatments that will enhance the effectiveness of decompression. In our DCM mouse model, excessive activation of the inflammatory response in the spinal cord has been associated with long-lasting symptoms and poor functional outcomes after decompression [7]. Interestingly, at 5 weeks after decompression in the present study, the number of white blood cells from the adaptive and innate immune system was significantly decreased. Although the systemic changes associated with decompression are different compared with other central nervous system (CNS) injury models, such as SCI, stroke, or multiple sclerosis (MS) [32–36]; a decreased number of T cell subsets has been reported as part of the normal response after major surgeries in patients [37]. In the context of traumatic SCI, mixed results associated with MP usage have led to significant controversy. When administered within the first 8 h after traumatic SCI, MP has been reported to improve neurological outcomes and short term motor scores [9, 38, 39]. However, aggregate evidence from different studies suggests a lack of effect in long-term motor recovery [39]. Despite evidence suggesting that steroids compromise the composition of the peripheral immune system in non-injured as well as injured conditions [24, 40–42], which raises concerns of possible negative side effects, the current guidelines for the management of acute SCI recommends MP treatment within the first 8 h of injury [8].

We have previously shown that there is an increased inflammatory response following delayed decompression for DCM, which can reduce the beneficial effects of surgical decompression [6, 7]. In the present study, MP administration resulted in, overall, small effects on the production of inflammatory cytokines, recruitment of Iba1^+^ cells and astrogliosis within the spinal cord. Albeit modest, these changes could lead to a more permissive environment that will allow neuronal preservation and an improved rate of functional recovery. This was reflected by an increased number of neurons in the MP-treated group, as compared to saline-treated animals. Similarly, in experimental models of traumatic SCI and ischemic optic neuropathy, MP treatment has been shown to preserve the number of neurons from apoptosis [43] and to reduce inflammation early after injury [11, 44]. Such regimens have been shown to accelerate the recovery of blood barrier integrity, reduce tissue damage, and attenuate recruitment of macrophages into the injured tissue [11, 44, 45]. MP may also be directly acting over neurons and their networks, potentially attenuating axonal excitability loss through the 5-HT_1A_ receptor [46]. These receptors are key players of the locomotor network in vertebrates responsible for a regular locomotor pattern, whose function that can be inhibited by the production of nitric oxide [47]. Similarly, patients receiving corticosteroids for lumbar decompression or cervical radiculopathy have been shown to experience a shorter duration of postoperative hospitalization and pain [12] [13]. In our model, MP effects on the inflammatory response were modest and did not translate to long-term gait improvement. This could be partially explained by the short half life of MP [48] or the frequency of MP delivery. Future studies will need to address whether a repeated low-dose MP injection protocol will be able to induce long-term gait improvements and assess the effects of such a protocol on the peripheral immune response.

Our study has certain limitations that should be acknowledged. Firstly, although mouse models are commonly used in research, the composition of peripheral white blood cells between humans and mice is different. Human white blood cells are enriched in granulocytes and monocytes, whereas mouse white blood cells are rich in B and T cells [49]. Therefore, the inflammatory response following surgical decompression for DCM may differ between mice and humans. Secondly, DCM patients may have other co-morbidities, including cardiovascular disease and diabetes, which are not present in our animal model. Thus, the potential clinical translation of this work to DCM patients will need to control for other potential side effects of steroids.

Importantly, the incidence of motor complications was reduced after decompression with MP treatment. Given that 1 out of 3 (34.9%) [50] patients undergoing decompression for DCM can develop postoperative complications, which are not only neurological in nature, the use of complementary treatments for decompression, such as MP, that can reduce this incidence rate are encouraged.

## Conclusions

In conclusion, the current study provides a deeper understanding of the peripheral immunological response following delayed decompression, and a detailed assessment of the role of MP in locomotor recovery following delayed surgical decompression. Larger studies, including prospective controlled trials in DCM patients, will be needed to better understand MP effects on the incidence of complications and enhancement of locomotor recovery, as well as the potential use of other anti-inflammatory drugs.

## Abbreviations

5-HT_1A_: 5-Hidroxitriptamina 1A
CNS: Central nervous system
DAPI: 4′,6-Diamidino-2-phenylindole
DCM: Degenerative cervical myelopathy
EDTA: Ethylenediaminetetraacetic acid
FDA: Food and Drug Administration
GFAP: Glial fibrillary acidic protein
Iba1: Ionized calcium binding adaptor molecule 1
IL-10: Interleukin-10
IL-1α: Interleukin 1-alpha
IL-1-β: Interleukin 1-beta
IL-4: Interleukin-4
IL-6: Interleukin-6
IRI: Ischemia-reperfusion injury
MP: Methylprednisolone
MS: Multiple sclerosis
NeuN: Neuronal nuclear protein
Olig-2: Oligodendrocyte transcription factor 2
PBS: Phosphate-buffered saline
PFA: Paraformaldehyde
SCI: Spinal cord injury
SEM: Standard error of the mean
TDA: Total Doppler area
TNF-α: Tumor necrosis factor-alpha
